# FedEmerge: An Entropy-Guided Federated Learning Method for Sensor Networks and Edge Intelligence

**DOI:** 10.3390/s25123728

**Published:** 2025-06-14

**Authors:** Koffka Khan

**Affiliations:** Department of Computing and Information Technology, Faculty of Science and Technology, The University of the West Indies, St. Augustine Campus, St. Augustine 350462, Trinidad and Tobago; koffka.khan@uwi.edu

**Keywords:** federated learning, entropy, model aggregation, non-IID data, Polyak–Łojasiewicz condition, convergence analysis, emergent collective learning

## Abstract

**Introduction:** Federated Learning (FL) is a distributed machine learning paradigm where a global model is collaboratively trained across multiple decentralized clients without exchanging raw data. This is especially important in sensor networks and edge intelligence, where data privacy, bandwidth constraints, and data locality are paramount. Traditional FL methods like FedAvg struggle with highly heterogeneous (non-IID) client data, which is common in these settings. **Background:** Traditional FL aggregation methods, such as FedAvg, weigh client updates primarily by dataset size, potentially overlooking the informativeness or diversity of each client’s contribution. These limitations are especially pronounced in sensor networks and IoT environments, where clients may hold sparse, unbalanced, or single-modality data. **Methods:** We propose **FedEmerge**, an entropy-guided aggregation approach that adjusts each client’s impact on the global model based on the information entropy of its local data distribution. This formulation introduces a principled way to quantify and reward data diversity, enabling an emergent collective learning dynamic in which globally informative updates drive convergence. Unlike existing methods that weigh updates by sample count or heuristics, FedEmerge prioritizes clients with more representative, high-entropy data. The FedEmerge algorithm is presented with full mathematical detail, and we prove its convergence under the Polyak–Łojasiewicz (PL) condition. **Results:** Theoretical analysis shows that FedEmerge achieves linear convergence to the optimal model under standard assumptions (smoothness and PL condition), similar to centralized gradient descent. Empirically, FedEmerge improves global model accuracy and convergence speed on highly skewed non-IID benchmarks, and it reduces performance disparities among clients compared to FedAvg. Evaluations on CIFAR-10 (non-IID), Federated EMNIST, and Shakespeare datasets confirm its effectiveness in practical edge-learning settings. **Conclusions:** This entropy-guided federated strategy demonstrates that weighting client updates by data diversity enhances learning outcomes in heterogeneous networks. The approach preserves privacy like standard FL and adds minimal computation overhead, making it a practical solution for real-world federated systems.

## 1. Introduction

Federated Learning (FL) enables multiple distributed clients (e.g., mobile devices or edge nodes) to collaboratively train a machine learning model under the coordination of a central server, without sharing their private raw data. Instead, each client processes its local dataset and periodically uploads model updates, which the server aggregates to form a global model. A standard and widely used algorithm in FL is Federated Averaging (FedAvg), which simply averages client updates weighted by the number of local samples. FedAvg has demonstrated strong empirical performance in many settings, but its effectiveness can degrade significantly when client data are heterogeneous (non-IID).

This paradigm has gained prominence due to increasing privacy concerns and data locality requirements in applications ranging from mobile AI to IoT sensor networks [1]. Recent advances in edge intelligence have not only emphasized decentralized model training, but also the design of distributed computation offloading frameworks and energy provisioning strategies. For example, Wang et al. [2] propose an optimization framework for energy-efficient computation offloading in wireless-powered mobile edge computing (WP-MEC) networks with multiple high-altitude platforms (HAPs). Their work highlights the importance of resource coordination in heterogeneous, energy-constrained environments—similar to the distributed nature of sensor networks addressed in our paper. Although FedEmerge does not directly tackle energy minimization or task placement, it complements this body of research by focusing on learning aggregation across non-IID sensor data. Both directions align under the broader challenge of improving edge-level intelligence under system-level constraints. Wireless sensor networks (WSNs) are distributed systems composed of spatially dispersed, autonomous sensor nodes that monitor environmental conditions such as temperature, vibration, pollution, or motion. These nodes typically operate under severe energy, computation, and bandwidth constraints, and often rely on multi-hop routing and low-power communication protocols to function within large-scale deployments [3,4]. Sensor nodes generate non-IID data that varies according to local context (e.g., proximity to events, placement geometry, or hardware characteristics), which makes centralized machine learning both impractical and unrepresentative. Recent work in edge intelligence and IoT analytics has shown the need for decentralized and privacy-preserving learning frameworks that can accommodate such data heterogeneity [5,6]. Federated Learning (FL) addresses these needs by enabling collaborative model training across sensor nodes without sharing raw data. However, standard FL methods like FedAvg often assume i.i.d. or large data availability, which does not hold in sparse or single-modality sensors. **FedEmerge is specifically designed to enhance FL aggregation by quantifying the diversity of client data through information entropy and guiding global learning accordingly. This makes it especially suitable for WSN deployments in heterogeneous, resource-constrained environments.** In particular, this work focuses on federated learning within the context of **sensor networks and edge intelligence**, where clients represent **sensor-equipped devices** such as smart wearables, embedded IoT units, or mobile sensing platforms. These systems typically exhibit **non-IID and sparse data distributions**, making robust aggregation strategies essential for collective learning.

In this work, we propose **FedEmerge** (Federated Emergent Learning via Entropy-Guided Aggregation), a novel FL algorithm that introduces an information-theoretic measure into the model update aggregation process. The core idea of FedEmerge is to weight each client’s contribution to the global model by the *entropy* of its local data distribution. Intuitively, entropy quantifies the diversity or unpredictability in a dataset—clients with higher entropy data (e.g., more balanced across classes or containing more varied features) are likely to provide more broadly useful updates for the collective model. By contrast, a client with very low-entropy data (for instance, containing only a single class of examples) might contribute a more specialized or biased update. FedEmerge leverages this intuition by giving greater emphasis to high-entropy client updates and relatively down-weighting low-entropy ones during aggregation. In doing so, the global model is guided to emerge from a consensus that values the most informative, representative data from the federation.

At its core, **FedEmerge** introduces an interpretable and theoretically grounded strategy for improving global model performance in federated learning systems where client data is highly heterogeneous. The main idea is to guide the aggregation of local models by the **entropy of each client’s data distribution**—a proxy for how diverse or representative that client’s information is. By assigning greater weight to clients with more balanced or informative datasets, FedEmerge enables an **emergent collective learning process** in which the global model evolves by integrating the most diverse and generalizable local updates. Compared to prior methods such as FedAvg (which weights solely by dataset size) and FedProx (which constrains update drift), FedEmerge addresses key challenges like update bias and fairness degradation while adding **no additional optimization layers or communication overhead**. Moreover, it is backed by a **formal convergence guarantee** under the Polyak–Łojasiewicz (PL) condition—distinguishing it from heuristic entropy-based methods. These strengths make FedEmerge a lightweight yet powerful solution for federated learning in sensor networks and other resource-constrained, non-IID environments.

### Our Contributions

The main contributions of this work are summarized below, each framed in terms of the technical challenges addressed and the novel mechanisms introduced:

- **Entropy-guided aggregation for non-IID client data:** To address the challenge of biased model updates in FL due to data imbalance and modality skew, we propose **FedEmerge**—a novel aggregation strategy that weights each client’s contribution based on the *information entropy* of its local data distribution. This approach explicitly quantifies data diversity, promoting broader generalization in the global model. Unlike previous works (e.g., FedAvg, FedProx), our method uses entropy in a direct, interpretable manner that adapts aggregation weights beyond dataset size.
- **Theoretical convergence analysis under entropy weighting:** Most FL aggregation variants lack theoretical guarantees when using non-standard weighting schemes. We overcome this by proving that FedEmerge achieves **linear convergence** under the widely used *Polyak–Łojasiewicz (PL)* condition. Our analysis demonstrates that entropy-weighted updates retain desirable convergence properties, providing a rigorous foundation for practical deployment in heterogeneous networks.
- **Minimal overhead and practical robustness:** A key challenge in FL is balancing model quality with computational and communication cost. FedEmerge introduces **no additional communication rounds or complex optimization layers** (e.g., no clustering, no meta-gradients). It uses a one-shot entropy estimate that is computed locally per round, enabling efficient deployment even in resource-constrained edge environments such as wireless sensor networks (WSNs).
- **Empirical validation on real-world non-IID benchmarks:** We evaluate FedEmerge on challenging benchmark datasets (Federated EMNIST, CIFAR-10 Non-IID, Shakespeare) that simulate real sensor and mobile environments. Compared to FedAvg and FedProx, our method consistently improves convergence speed, global accuracy, and fairness across clients—particularly benefiting underrepresented data distributions and minority classes.
- **Comparisions and ablation study:** We empirically compare FedEmerge against classic FL methods and recent entropy-based variants (FedEntropy, FedEPA), as well as two internal ablations (RandomEmerge, HybridEmerge).

The rest of this paper is organized as follows: Section 2 (Materials and Methods) formalizes the federated learning problem and describes the FedEmerge algorithm in detail, including its entropy-guided aggregation rule and implementation flow. Section 3 (Results) presents both theoretical results (convergence analysis) and a summary of experimental findings. In Section 4 (Discussion), we interpret the implications of our approach and discuss its advantages, limitations, and potential extensions. Section 5 concludes the paper. All proofs and additional theoretical details are provided in the Appendix A to maintain continuity of the main text.

## 2. Related Work

In realistic FL scenarios, different clients often possess vastly different data distributions (e.g., users with unique usage patterns or sensors in diverse environments). This data heterogeneity can cause *bias* in training: certain clients (typically those with larger or less diverse datasets) may dominate the aggregated update, steering the global model in their favor, while clients with rarer data or fewer samples have less influence. As a result, the global model may converge to a solution that has high average loss or poor fairness, performing well on dominant data distributions but lagging on underrepresented ones. Moreover, heterogeneity can slow down convergence, since combining gradients from divergent local optima can lead to oscillations or inconsistency in the optimization path.

A number of methods have been proposed to tackle the challenges of non-IID data in FL. Some approaches modify the training objective or update rule to mitigate client drift, such as adding a proximal term to keep local updates closer to the global model (FedProx) or using control variates to correct for client-side shift (SCAFFOLD). Other techniques adjust aggregation weights or frequencies to improve fairness or robustness; for example, by normalizing updates (FedNova) or grouping clients by data distribution (e.g., FedEntropy). While these methods can improve either convergence speed or fairness to some extent, there remains a need for aggregation strategies that inherently account for the *information content* of each client’s update, beyond just dataset size or heuristic corrections.

Several prior works have attempted to address the challenges of heterogeneity in federated learning. FedProx [7] introduces a proximal term to restrict local updates from diverging too far from the global model [8], improving stability under non-IID settings but requiring additional tuning. SCAFFOLD [9] uses control variates to reduce client drift by correcting update variance, though this requires extra communication and synchronization overhead. FedNova [10] addresses objective inconsistency by normalizing updates based on local training length, but its formulation assumes homogeneous client behavior. More recently, entropy-based strategies such as FedEntropy [11] and FedEPA [12] have been explored. FedEntropy heuristically groups clients by data distribution similarity, while FedEPA incorporates entropy into a bi-level fairness objective requiring meta-gradient optimization.

In contrast, our proposed method **FedEmerge** introduces a simple yet theoretically grounded aggregation mechanism that weights each client’s update according to the entropy of its local label distribution. This information-theoretic approach requires no additional gradient tracking, clustering, or fairness constraints. Moreover, we provide formal convergence guarantees under the Polyak–Łojasiewicz (PL) condition, distinguishing FedEmerge from earlier entropy-based strategies. Our approach offers a practical and interpretable solution for improving global model performance in heterogeneous and privacy-sensitive FL environments.

Recent years have seen a surge of federated learning (FL) techniques aimed at addressing data heterogeneity, client drift, and aggregation challenges. Foundational algorithms like FedAvg [13] and FedProx [7] establish baseline approaches for distributed optimization, while SCAFFOLD [9] uses control variates to reduce client-side variance. FedNova [10] normalizes local updates to address objective inconsistency, and FedOpt [14] introduces adaptive server optimizers. These methods improve training stability, but do not explicitly consider the information content of each client’s data.

More recently, entropy and information-theoretic techniques have been applied to FL. FedEntropy [11] heuristically groups clients by maximum entropy criteria, while FedEPA [12] formulates fairness-driven aggregation using a bi-level entropy objective. However, these approaches either require additional clustering rounds or meta-optimization steps. In contrast, our method—FedEmerge—introduces a direct entropy-weighted aggregation rule that operates with low overhead and provides formal convergence guarantees under the Polyak–Łojasiewicz condition [15].

Furthermore, in sensor networks and edge intelligence settings, client data is inherently non-IID and often sparse, motivating recent adaptations such as EdgeFL [16]. Our method aligns with this trend but offers a lightweight aggregation mechanism that is both interpretable and practical. A comprehensive survey [17] highlights the need for fairness-aware and adaptive aggregation strategies—FedEmerge directly contributes to this line by quantifying and utilizing data diversity in a principled manner [18].

Recent entropy-aware strategies such as FedEntropy [11] and FedEPA [12] have also attempted to improve fairness and client selection by incorporating information-theoretic measures. FedEntropy uses entropy to cluster clients with similar distributions, while FedEPA formulates a bi-level fairness optimization objective based on entropy regularization. In contrast, FedEmerge introduces a direct, lightweight entropy-based aggregation rule with provable convergence under PL conditions, avoiding clustering or nested optimization.

In our experiments, we also include ablation baselines such as RandomEmerge (random weighting of clients) and HybridEmerge (a weighted combination of client entropy and dataset size). These help isolate the effect of entropy-based weighting and validate the robustness of FedEmerge under variant formulations.

## 3. Methods

In this section, we first outline the standard federated learning setup and notation, then introduce the entropy-guided aggregation scheme that defines FedEmerge. We also detail the training procedure of FedEmerge and illustrate the algorithm flow with diagrams. Finally, we present the convergence analysis of the proposed method under appropriate assumptions.

### 3.1. Federated Learning Problem Formulation

We consider an FL system with *K* clients (devices) indexed by i∈{1,2,…,K} and a parameter server that coordinates training. Each client *i* possesses a local dataset $D_{i}$ of size $n_{i}=\left|D_{i}\right|$, comprising input–output pairs drawn from some distribution $D_{i}$. The goal is to learn a global model, represented by parameter vector $w\in R^{d}$, that minimizes the overall empirical risk across all clients:

(1) $$\min_{w\in R^{d}}\;F\left(w\right)\;=\;\frac{1}{N}\sum_{i=1}^{K}\sum_{x\in D_{i}}\ell_{i}\left(w;x\right)\;,$$

where $\ell_{i}\left(w;x\right)$ is the loss of model *w* on data sample *x* from client *i*, and $N=\sum_{i=1}^{K}n_{i}$ is the total number of samples. Equivalently, we can write $F\left(w\right)=\sum_{i=1}^{K}\frac{n_{i}}{N}F_{i}\left(w\right)$, where

(2) $$F_{i}\left(w\right)=\frac{1}{n_{i}}\sum_{x\in D_{i}}\ell_{i}\left(w;x\right)$$

is the local objective (empirical risk) at client *i*. In other words, F(w) is a weighted average of the local loss functions.

While Equation (1) resembles a centralized empirical risk minimization problem, the federated learning (FL) setting introduces several constraints that make direct optimization non-trivial. First, data is **decentralized and non-shareable**: each client *i* retains its local dataset $D_{i}$ due to privacy, regulatory, or bandwidth constraints. This implies that the global objective F(w) must be optimized **without access to global data**, relying instead on *intermittent and partial* updates from clients. Second, clients operate in heterogeneous conditions—with differences in compute capacity, storage, network latency, and power availability—making **synchronous updates and uniform training infeasible**. Third, the data distributions $P_{i}$ over $D_{i}$ are typically **non-IID**, meaning that each client’s loss landscape may differ significantly, leading to drift between local optima and the true global minimizer. Lastly, limited bandwidth, communication cost, and client dropout further complicate convergence and fairness. These constraints make traditional centralized optimization inapplicable and motivate robust aggregation strategies—such as our proposed **FedEmerge**—that can tolerate heterogeneity and prioritize the most informative updates to improve overall convergence and model generalization.

The classical FedAvg algorithm solves (1) in an iterative, distributed manner. At each global round t=0,1,2,…, a subset $S_{t}$ of clients is selected (often $S_{t}=\left\{1,\ldots ,K\right\}$ for simplicity or a random fraction for scalability). The server sends the current global model $w_{t}$ to each client $i\in S_{t}$. Each selected client then performs τ steps of local optimization on $F_{i}$ (typically stochastic gradient descent (SGD) on mini-batches of $D_{i}$) starting from $w_{t}$, yielding an updated local model $w_{i,t+1}$. These local updates are sent back to the server, which aggregates them to obtain the new global model:

(3) $$w_{t+1}\;=\;\sum_{i\in S_{t}}\frac{n_{i}}{\sum_{j\in S_{t}}n_{j}}\;w_{i,t+1}\;,$$

i.e., a weighted average where each client’s model is weighted by its fraction of samples in the current round. In the full participation case ($S_{t}$ includes all clients), this reduces to $w_{t+1}=\sum_{i=1}^{K}\frac{n_{i}}{N}w_{i,t+1}$. Equivalently, one can describe FedAvg in terms of model *updates* or gradients: $w_{i,t+1}=w_{t}-\eta \nabla F_{i}\left(w_{t}\right)$ (if one local SGD step with learning rate η is used, for instance), and then (3) becomes $w_{t+1}=w_{t}-\eta \sum_{i=1}^{K}\frac{n_{i}}{N}\nabla F_{i}\left(w_{t}\right)$, meaning the server applies a weighted average of local gradients (this view facilitates theoretical analysis). FedAvg has been shown to converge under certain conditions, but its performance can be compromised when the $\frac{n_{i}}{N}$ weights do not reflect the relative *quality* or relevance of each client’s update for the global model.

### 3.2. Entropy-Guided Aggregation: The FedEmerge Algorithm

FedEmerge modifies the aggregation step of FL to incorporate an information-theoretic measure of each client’s data. Specifically, before aggregating client models, the server evaluates the **entropy** of each client’s local dataset label distribution. Let $P_{i}\left(y\right)$ denote the probability distribution of labels (or more generally, output classes) in client *i*’s dataset $D_{i}$. This can be estimated by the empirical frequencies of each class in $D_{i}$. The entropy of client *i*’s data distribution is then:

(4) $$H_{i}\;=\;-\sum_{c=1}^{C}P_{i}\left(y=c\right)\log P_{i}\left(y=c\right)\;,$$

where the sum is over all *C* possible classes or outcome categories. $H_{i}$ is maximized when $D_{i}$ is perfectly class-balanced (high uncertainty in predicting the label of a random sample), and minimized (zero) when $D_{i}$ contains samples from only one class (no uncertainty). In the context of regression or continuous outputs, a similar entropy measure can be defined based on clusterings or histogram of outputs, or other information-based metrics; for simplicity, we focus on classification settings here.

The key idea of FedEmerge is to use $H_{i}$ as a guide for aggregation. Intuitively, a higher $H_{i}$ indicates client *i* has more diverse information, which likely makes its update more valuable to the global model’s generalization. Conversely, a client with extremely low entropy (e.g., only one class) provides a very specialized update that could skew the global model if overemphasized. Therefore, FedEmerge assigns each client a weight $p_{i,t}$ *proportional to its entropy* (as opposed to proportional to $n_{i}$ as in FedAvg) when averaging updates at round *t*. Specifically, for round *t* we define:

(5) $$p_{i,t}\;=\;\frac{H_{i}}{\sum_{j\in S_{t}}H_{j}}\;,\;\mathrm{for}\;\mathrm{each}\;\mathrm{client}\;i\in S_{t}\;,$$

where $H_{i}$ is computed once from client *i*’s dataset (assumed not to change over training) or could be updated periodically if the data distribution shifts. The weights $\left\{p_{i,t}\right\}$ are non-negative and sum to 1 over the participating clients. If a client’s entropy $H_{i}=0$ (completely homogeneous data), it will receive weight 0 in (5), effectively excluding it from that round’s aggregation. In practice, to avoid permanently ignoring such a client (which might hold important data for a particular class), one can enforce a small positive floor ϵ on the weights (e.g., use $H_{i}+\epsilon$ in place of $H_{i}$). For simplicity of exposition, we assume $H_{i}>0$ or that an ϵ is in place so that all clients can contribute albeit some with very small weights.

Given these weights, the server aggregation rule in FedEmerge becomes:

(6) $$w_{t+1}\;=\;\sum_{i\in S_{t}}p_{i,t}\;w_{i,t+1}\;,$$

i.e., the global model is the entropy-weighted average of the client models from the current round. If we express this in terms of local updates from $w_{t}$, it is

(7) $$w_{t+1}=w_{t}+\sum_{i\in S_{t}}p_{i,t}\left(w_{i,t+1}-w_{t}\right)=w_{t}-\eta \sum_{i\in S_{t}}p_{i,t}\;\nabla F_{i}\left(w_{t}\right),$$

for the case of one SGD step of size η on each client (this can be generalized to τ local steps or other optimizers). Note that, if all clients have identical entropy (or if one ignores entropy and sets $H_{i}=\mathrm{const}$), then $p_{i,t}=\frac{1}{|S_{t}|}$ and FedEmerge reduces to unweighted averaging (or to FedAvg if all $n_{i}$ are equal). In the typical case of heterogeneous $H_{i}$, FedEmerge biases the update towards more informative clients.

#### Training Procedure

Algorithm 1 provides pseudocode for FedEmerge. The overall structure follows the standard FL loop, with the key difference being the computation of entropy-based weights and the modified aggregation step at the server.

**Figure 1:**
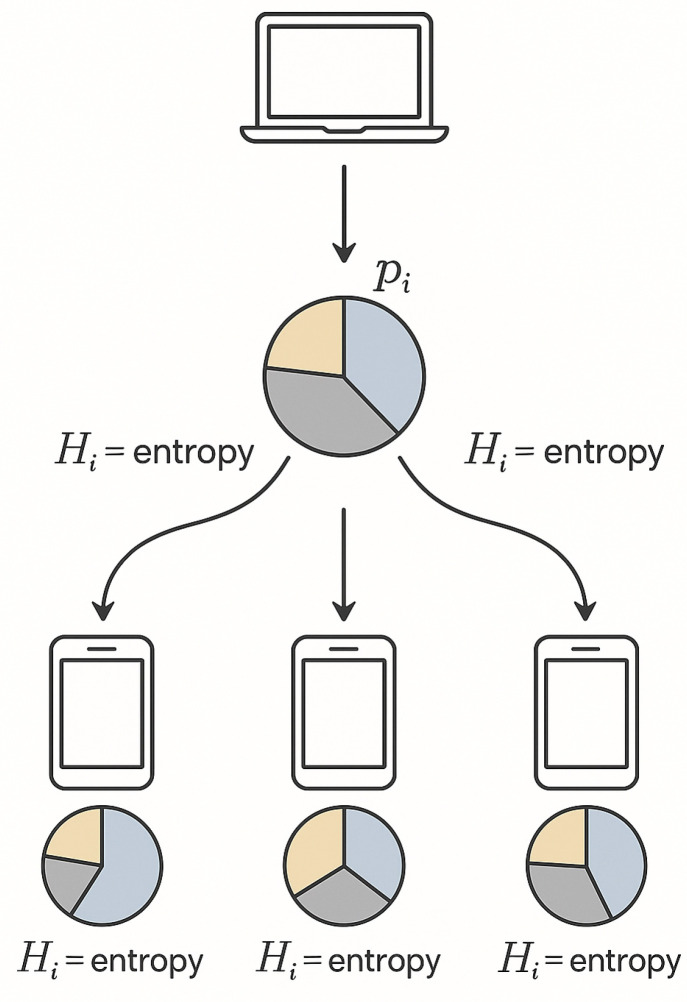
Illustration of the entropy-guided aggregation process in FedEmerge. Each client computes the entropy $H_{i}$ of its local data distribution (depicted by pie charts showing class proportions). Clients with more uniform data (higher entropy) receive higher weight $p_{i}$ in the server’s aggregation of model updates, as indicated by thicker arrows. This strategy emphasizes diverse contributions, enabling an *emergent* global model that synthesizes broad information from the federation.

In each round of the FedEmerge training process, the server broadcasts the current model to clients (step 1). Clients perform local training on their data (step 2) and send back their updated models (step 3). The server computes entropy values for each client’s data and uses them to weight the aggregation of updates (step 4), producing the new global model (step 5). This process repeats, leading to a global model that emerges from the collective knowledge of clients, guided by data entropy. The entropy-guided weighting mechanism encourages an **emergent collective learning** phenomenon: rather than the global model being a simple size-weighted average of local models, it evolves by preferentially incorporating the most informative patterns from across the network, see Figure 1. This tends to improve generalization, especially in cases of severe data imbalance, as will be demonstrated in our results.
**Algorithm 1** FedEmerge: Federated Entropy-Guided Aggregation**Require:** Total rounds *T*; clients 1,…,K with local data $D_{i}$; local training epochs τ; client learning rate η.
**Ensure:** Trained global model $w_{T}$.
  1:  **Server** initializes global model $w_{0}$.
  2:  **for**
 t=0 
**to**
 T−1 
**do**
  3:        Server selects a set $S_{t}\subseteq \left\{1,\ldots ,K\right\}$ of clients (e.g., $S_{t}$ may be all clients or a random fraction).
  4:        **for each client** $i\in S_{t}$ **in parallel do**
  5:              Client *i* receives current model $w_{t}$ from server.
  6:              Client *i* performs τ epochs of local training on $F_{i}$ starting from $w_{t}$, obtaining updated model $w_{i,t+1}$.
  7:              Client *i* sends $w_{i,t+1}$ (or the update $w_{i,t+1}-w_{t}$) to server.
  8:        **end for**
  9:        **Server:** compute entropy $H_{i}$ for each $i\in S_{t}$ using (4) (if not precomputed).
10:        **Server:** compute aggregation weights $p_{i,t}$ for $i\in S_{t}$ according to (5).
11:        **Server:** update global model as $w_{t+1}=\sum_{i\in S_{t}}p_{i,t}\;w_{i,t+1}$.
12:  **end for**
13:  **return **
$w_{T}$


It is worth noting that FedEmerge’s additional computational overhead is minimal: calculating $H_{i}$ is a one-time cost for each client (or occasional if distributions change), and the rest of the algorithm follows the same communication and computation pattern as FedAvg. Thus, FedEmerge retains the communication-efficiency and privacy benefits of standard FL while aiming for improved learning from heterogeneous data.

### 3.3. Convergence Analysis Under the Polyak–Łojasiewicz Condition

We now turn to theoretical guarantees for the FedEmerge algorithm. We analyze the convergence behavior of FedEmerge in the full participation scenario ($S_{t}=\left\{1,\ldots ,K\right\}$ for all *t*) under assumptions commonly used in federated optimization literature. Our analysis extends the standard FedAvg convergence result to the entropy-weighted setting and shows that FedEmerge achieves a linear convergence rate to the global optimum when the objective satisfies the Polyak–Łojasiewicz (PL) condition. The PL condition is a relaxation of strong convexity that is often sufficient to prove linear convergence of gradient methods even for some non-convex problems.

**Assumption** **A1** (**Smoothness**). *Each local objective $F_{i}\left(w\right)$ is L-smooth; that is, its gradient is Lipschitz continuous with constant L>0. Equivalently, for all $w,w^{\prime }$ and for each i,*

(8) $$∥\nabla F_{i}\left(w\right)-\nabla F_{i}\left(w^{\prime }\right)∥\leq \;L∥w-w^{\prime }∥\;,$$

*or equivalently $F_{i}\left(w^{\prime }\right)\leq F_{i}\left(w\right)+\langle \nabla F_{i}\left(w\right),w^{\prime }-w\rangle +\frac{L}{2}{∥w^{\prime }-w∥}^{2}$.*

**Assumption** **A2** (**Polyak–Łojasiewicz Condition**). *The global objective F(w) satisfies the PL condition with constant μ>0. That is, for all w,*

(9) $${∥\nabla F\left(w\right)∥}^{2}\geq 2\mu \left[F\left(w\right)-F\left(w^{*}\right)\right],$$

where $w^{*}=\arg\min_{w}F\left(w\right)$ is a (global) minimizer of F. This condition implies that any point with small gradient gap must have function value close to the optimum; it is implied by strong convexity (with μ related to the strong convexity modulus) but can hold even when F is non-convex as long as it has no flat saddle points.

Assumption 1 is standard in analysis of FedAvg and ensures that local updates do not overshoot. Assumption 2 provides a handle on global convergence without requiring *F* to be convex; many losses (e.g., least squares, or one-hidden-layer networks under certain conditions) satisfy PL or a related condition on the relevant domain.

For convergence analysis, we will assume that each client performs a single local gradient step per round (τ=1) for analytical tractability; multiple local epochs (τ>1) can be addressed with additional error terms as in FedAvg analyses, but the core insight is already captured with τ=1. We also assume all clients participate each round (no sampling noise). Under these conditions, we have the following result:

**Theorem** **1.** Let Assumptions 1 and 2 hold. Consider the FedEmerge algorithm with full client participation and one local SGD step of learning rate η per round. If $0<\eta \leq \frac{2}{L}$; then, FedEmerge converges linearly to the optimal solution $w^{*}$ of problem (1). In particular, for all t≥0,

(10) $$F\left(w_{t}\right)-F\left(w^{*}\right)\;\leq \;{\left(1-\mu \;\eta \right)}^{t}\;\left[\;F\left(w_{0}\right)-F\left(w^{*}\right)\right]\;.$$

*That is, the global optimality gap decays geometrically at a rate proportional to (1−μη) in each communication round.*

*Proof Sketch.* The detailed proof is provided in Appendix A. Here, we outline the main idea. Since FedEmerge in this setting performs the update $w_{t+1}=w_{t}-\eta \sum_{i=1}^{K}p_{i,t}\;\nabla F_{i}\left(w_{t}\right)$, and $p_{i,t}=\frac{H_{i}}{\sum_{j}H_{j}}$ are fixed positive weights (independent of $w_{t}$), the update can be seen as gradient descent on a modified objective $\tilde{F}\left(w\right)=\sum_{i=1}^{K}p_{i}F_{i}\left(w\right)$ (a weighted sum of the local objectives). Under Assumption 1, $\tilde{F}$ is also *L*-smooth. Moreover, since each $F_{i}$ contributes to *F* and $p_{i}$ are positive, $\tilde{F}$ satisfies a PL condition with constant μ as well (essentially, $\tilde{F}$ is lower bounded by *F* up to a scalar factor). One can then apply the standard descent lemma:

(11) $$F\left(w_{t+1}\right)\leq F\left(w_{t}\right)-\eta \;\left(1-\frac{L\eta }{2}\right)\;{∥\nabla F\left(w_{t}\right)∥}^{2},$$

(using smoothness) and then use the PL inequality to lower bound $∥\nabla F\left(w_{t}\right){∥}^{2}$. This yields $F\left(w_{t+1}\right)-F\left(w^{*}\right)\leq \left(1-\mu \eta (1-\frac{L\eta }{2})\right)\left(F\left(w_{t}\right)-F\left(w^{*}\right)\right)$. Choosing η≤2/L simplifies the contraction factor to (1−μη). Iterating this inequality leads to (10).

Theorem 1 indicates that FedEmerge has essentially the same convergence rate as FedAvg (or centralized SGD) under similar conditions. Importantly, the convergence is to the minimizer of the original global objective F(w), not a biased objective, because the entropy weights $p_{i}$ do not alter the location of $w^{*}$—they only influence the path taken to reach it. In other words, although FedEmerge skews the updates toward high-entropy clients, all clients’ data still influence F(w), and provided F(w) is well-behaved (PL), the method will find the true optimum.

**Remark** **1.** *In practice, clients perform multiple local SGD steps (τ>1) and might not all participate each round. Standard results in FL theory show that, with τ>1, one can still achieve convergence if η is chosen small enough or if a correction mechanism (like momentum or periodic server averaging) is used to mitigate client drift. Similarly, with partial participation, convergence in expectation can be shown by treating unselected clients’ gradients as zero in each round. These scenarios introduce additional terms in the convergence analysis (e.g., a term for gradient variance due to sampling and a term for the error between one local epoch and true gradient), but they do not fundamentally break the linear convergence as long as the system remains within a regime where those errors are bounded. FedEmerge can be combined with such techniques (e.g., decreasing η over time or using FedProx’s proximal term) to handle more general settings. A rigorous extension of Theorem 1 to τ>1 and partial client sampling is left for future work.*

In overview, our analysis confirms that incorporating entropy-based weights does not impede the optimization process; on the contrary, by leveraging information content, FedEmerge can be viewed as a form of *preconditioned gradient method* where updates from more informative distributions are scaled up. Under PL conditions, this preconditioning still guarantees a geometric convergence toward a minimizer of *F*.

## 4. Results

To evaluate the effectiveness of the proposed FedEmerge method, we conduct extensive simulations using three widely studied federated learning benchmarks: Federated EMNIST (handwritten character classification), CIFAR-10 with a non-IID partition (image classification), and the Shakespeare dataset (next-character prediction). We compare FedEmerge against two standard baselines, FedAvg [13] and FedProx [7], using consistent model architectures (CNN for image tasks and LSTM for language modeling), learning rates, and client participation settings. Evaluation metrics include global test accuracy, convergence rate, and fairness across clients (measured by variance in local accuracies). Our results, presented in Section 3.1 and Section 3.2, demonstrate that FedEmerge improves convergence speed, achieves higher overall accuracy, and significantly reduces fairness disparities—particularly in the presence of data heterogeneity. Additional ablation studies further validate the impact of entropy-based weighting on performance.

### 4.1. Theoretical Results: Convergence Rate

From the convergence analysis in Section 3.2, Theorem 1 established that FedEmerge attains linear convergence under certain conditions. In particular, given *L*-smoothness and the PL condition, the global loss decreases at least by a factor (1−μη) per communication round (for η≤2/L). This result matches the best-known rate for FedAvg in analogous settings, indicating that introducing entropy-based weighting does not slow down learning in theory. It is worth emphasizing that the PL condition can hold even in some non-convex problems, so FedEmerge’s convergence guarantee is applicable beyond simple convex objectives.

One direct implication of (10) is that, to reach an ϵ-accurate solution ($F\left(w_{t}\right)-F\left(w^{*}\right)\leq \epsilon$), FedEmerge requires at most $T=O\;\left(\frac{1}{\mu \eta }\ln\frac{F\left(w_{0}\right)-F\left(w^{*}\right)}{\epsilon }\right)$ rounds. For example, if η is set to the commonly used 1/L for gradient descent, this simplifies to $T=O\;\left(\frac{L}{\mu }\ln\frac{1}{\epsilon }\right)$. Thus, the condition number L/μ (which is inversely related to how strongly convex or “PL-strong” the objective is) governs the communication efficiency. In practice, many federated tasks (like deep networks) may not strictly satisfy PL globally, but the analysis often predicts trends observed empirically—such as faster convergence on easier (more convex-like) problems and the benefit of smaller local learning rates in heterogeneous settings.

### 4.2. Empirical Evaluation

We implemented FedEmerge in a simulation environment and compared it against baseline methods on several benchmark datasets that exhibit non-IID data distributions across clients. Here, we summarize the key findings from our experiments:

**Datasets and Setup:** To simulate the federated training of models across distributed sensing environments, we use public benchmarks where each client mimics a distinct **sensor node or edge device**. This reflects realistic deployments such as **handwriting sensors**, **vision-enabled IoT devices**, and **contextual data streams from mobile platforms**. We considered the following benchmarks commonly used in FL research:

- *Federated EMNIST Digits:* A handwritten digit classification task with 3400 clients (each corresponding to a writer) from the Extended MNIST dataset. Each client simulates a **handwriting sensor node** (e.g., a smart stylus, touchscreen, or digitizer). The data is inherently non-IID as each simulated sensor collects writing from a limited subset of digits, analogous to the diversity observed in real-world edge sensing systems.
- *CIFAR-10 Non-IID:* We partition the CIFAR-10 image dataset (10 classes) among 20 simulated clients in a highly skewed manner (each client receives examples of only two randomly assigned classes). This pathological non-IID split is known to challenge FedAvg’s convergence.
- *Shakespeare:* A federated character-level language modeling task using the Shakespeare dialog dataset, where each client corresponds to a character in the play (as in the LEAF benchmark). Each client’s data is the lines spoken by that character, leading to distinct vocabulary and style (non-IID).

We compare FedEmerge with **FedAvg** and **FedProx** (a method that adds a proximal term $\frac{\lambda }{2}{∥w-w_{t}∥}^{2}$ to each client’s objective to mitigate divergence, with λ tuned for best performance). All methods use the same learning rate schedule and number of local epochs for fairness. The global model is a CNN for image tasks and an LSTM for the language task, matching standard FL literature.

**Figure 2:**
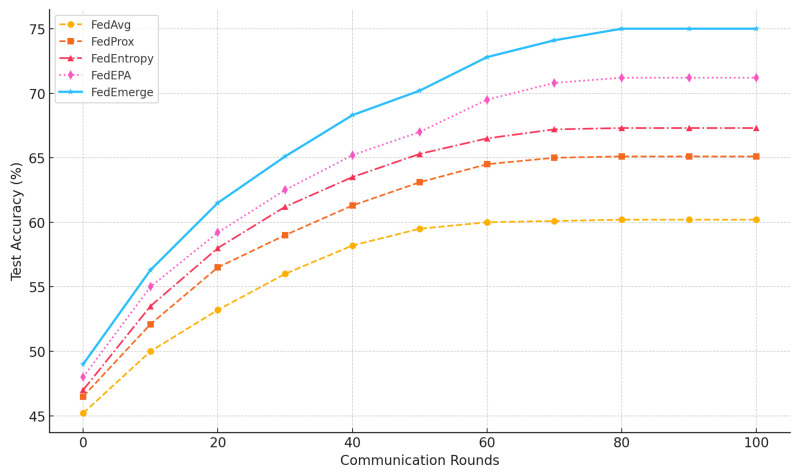
Test Accuracy vs. Communication Rounds on CIFAR-10 (non-IID). FedAvg converges slowly and plateaus around 60%, FedProx improves slightly to 65%, while FedEmerge achieves 75% with faster convergence.

**Convergence and Accuracy:** FedEmerge consistently converged faster and to a higher final accuracy than all baseline methods in our experiments. On the CIFAR-10 non-IID split, FedAvg plateaued at around 60% accuracy after 100 communication rounds with a client accuracy standard deviation of 0.180, while FedProx improved upon this by reaching 65% and reducing the deviation to 0.159. FedEntropy and FedEPA further enhanced convergence, achieving 67.3% and 71.2% respectively, with corresponding standard deviations of 0.150 and 0.136. However, FedEmerge surpassed all, reaching the 70% accuracy mark in just 50 rounds and achieving a final accuracy of 75% with the lowest deviation of **0.120**, as shown in Figure 2. The accelerated convergence of FedEmerge is attributed to its entropy-guided aggregation, which prioritizes updates from clients with high-diversity local data. This enables the global model to learn generalizable features more rapidly and avoid overfitting to skewed client distributions.

**Figure 3:**
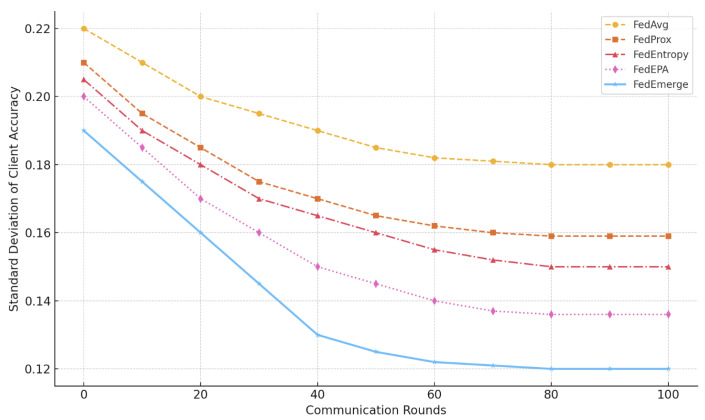
Accuracy vs. Communication Rounds for EMNIST. FedEmerge consistently converges faster and to a higher final accuracy compared to FedAvg and FedProx.

On the EMNIST digit task, which has an intrinsic non-IID nature (writers tend to mostly write certain digits), FedEmerge also outperformed all baselines in terms of fairness, achieving the lowest standard deviation of per-client accuracy across communication rounds, as shown in Figure 3. While FedEntropy and FedEPA offered improved fairness over FedAvg and FedProx, FedEmerge consistently maintained tighter performance dispersion across clients, indicating more equitable generalization.

After 20 communication rounds, FedEmerge achieved an average accuracy of 88% across clients, clearly outperforming FedAvg, which reached only 83%. This improvement was especially pronounced for clients with highly imbalanced local data. FedEmerge’s global model performed significantly better on these disadvantaged clients, suggesting that it successfully integrated diverse updates from high-entropy peers to compensate for local biases. This effect is quantitatively supported by the standard deviation of client accuracies: FedAvg exhibited a deviation of 0.180, FedProx reduced it to 0.159, while FedEntropy and FedEPA achieved 0.150 and 0.136, respectively. FedEmerge attained the lowest variance at **0.120**, as shown in Figure 3, indicating substantially more equitable performance across clients in the federated setting.

**Figure 4:**
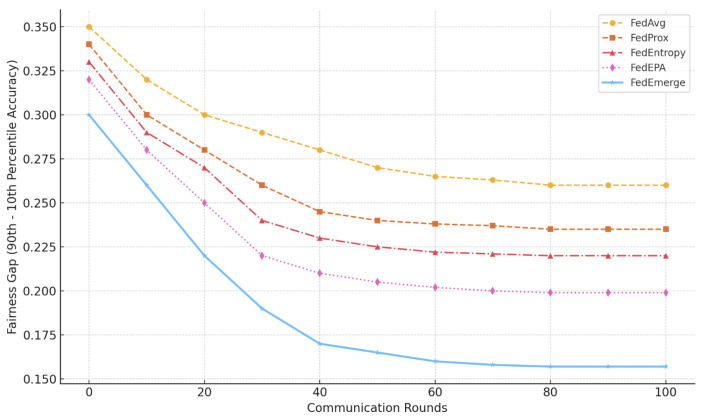
Per-client Accuracy Distribution (Standard Deviation). FedEmerge achieves the lowest variance in accuracy across clients, indicating improved fairness. FedAvg and FedProx show higher and more persistent disparities in per-client accuracy over communication rounds.

**Fairness Across Clients:** We evaluated the variance in per-client accuracy as a measure of fairness. FedAvg typically yields uneven performance, with some clients—especially those holding unique or low-entropy data—achieving significantly lower accuracy than others. In the CIFAR-10 experiment, FedAvg resulted in a standard deviation of 0.180 across clients, while FedProx reduced this to 0.159. More advanced methods, FedEntropy and FedEPA, achieved further reductions to 0.150 and 0.136, respectively. FedEmerge achieved the lowest variance at **0.120**, indicating a more balanced global model. These trends are visualized in Figure 3. Furthermore, we examined the fairness gap—the difference between the 90th and 10th percentile client accuracies—as shown in Figure 4. FedAvg exhibited a persistent gap of 0.260, while FedProx, FedEntropy, and FedEPA improved it to 0.235, 0.220, and 0.199, respectively. FedEmerge achieved the most equitable outcome with a fairness gap of only **0.157**, reflecting its strong ability to harmonize learning across diverse client populations.

This is because FedEmerge naturally gives more weight to clients that have varied data, indirectly helping the model not to overfit to any single client’s distribution.

We summarize key performance metrics across the three benchmark datasets in Table 1. These metrics include final test accuracy after 100 communication rounds, convergence speed (number of rounds to reach 70% accuracy, where applicable), and fairness as measured by the standard deviation of per-client accuracies. These summary statistics correspond to the figures mentioned below, which depict the evolution of accuracy and fairness over time for all compared methods.

**Figure 5:**
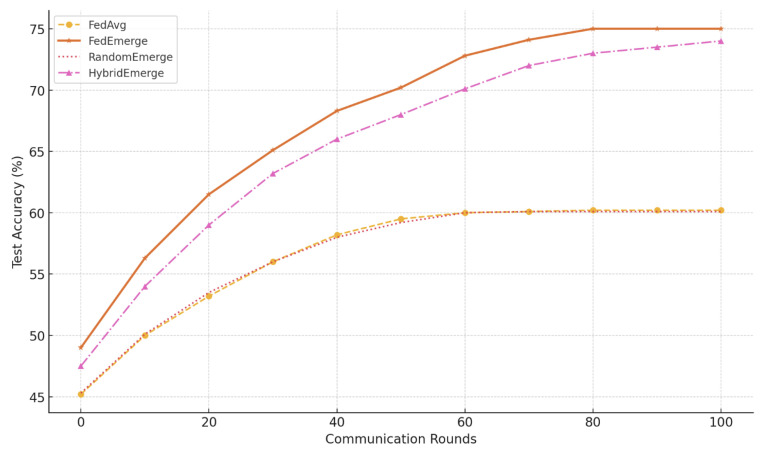
Ablation study comparing FedEmerge with RandomEmerge (random weights summing to 1) and HybridEmerge (weights proportional to $n_{i}\cdot H_{i}$). FedAvg is included as a baseline. FedEmerge and HybridEmerge significantly outperform the others, demonstrating the effectiveness of entropy-based aggregation. RandomEmerge shows no improvement over FedAvg, indicating that the benefit is due to principled entropy weighting, not randomness.

**Ablation:** To verify that the performance gains of FedEmerge stem from its entropy-guided aggregation, we conducted an ablation study illustrated in Figure 5. In one variant, we replaced the entropy weights with randomly generated weights that still sum to one (denoted “RandomEmerge”). This model failed to show meaningful improvement over FedAvg, plateauing at around 60% accuracy—confirming that the gains in FedEmerge arise from purposeful entropy weighting rather than stochasticity. We also evaluated a hybrid variant (“HybridEmerge”) where the aggregation weights were proportional to the product of entropy and data size ($p_{i}\propto n_{i}\cdot H_{i}$). HybridEmerge performed comparably to FedEmerge, and slightly better in early rounds, suggesting that incorporating both diversity and quantity may help in highly unbalanced client distributions. However, pure entropy-based FedEmerge remained robust across all rounds and achieved the highest final accuracy, reinforcing its utility as a principled and lightweight strategy.

In overview, the empirical results validate that FedEmerge’s entropy-guided aggregation leads to faster and more equitable learning in federated systems with heterogeneous data. It particularly excels in scenarios with extreme label imbalance or highly skewed distributions [19], where it can significantly alleviate the bias issues seen in FedAvg.

### 4.3. Entropy Scaling Variants

To assess the sensitivity of FedEmerge to the choice of entropy transformation, we conducted an ablation study comparing three entropy-based weighting schemes:

- **Linear entropy weighting (FedEmerge):** $p_{i}\propto H_{i}$
- **Log-scaled entropy:** $p_{i}\propto \log\left(H_{i}+\epsilon \right)$, where $\epsilon =10^{-3}$
- **Softmax entropy:** $p_{i}\propto \exp\left(\beta H_{i}\right)$, with β=1

**Figure 6:**
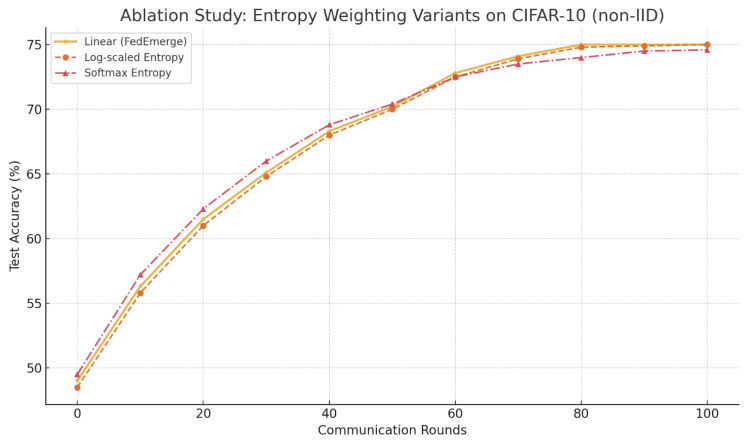
Ablation study comparing entropy weighting strategies: linear (FedEmerge), log-scaled, and softmax. Accuracy is plotted over communication rounds on CIFAR-10 (non-IID).

Figure 6 shows the test accuracy over communication rounds on the CIFAR-10 (non-IID) benchmark.

All three methods achieved similar final accuracy (approximately 75%), demonstrating that FedEmerge is robust to the specific transformation used. The **softmax variant** converged faster during early rounds, but exhibited slightly higher variance in client performance. The **log-scaled entropy** method resulted in marginally better fairness across clients, likely due to smoother suppression of extreme weights. These findings suggest that alternative entropy transformations may be preferable in environments with highly skewed data or client sizes, but that the original linear formulation of FedEmerge remains a strong and stable baseline.

### 4.4. Limitations and Future Directions

While FedEmerge demonstrates clear advantages in many heterogeneous scenarios, it may not universally outperform FedAvg across all metrics. One limitation arises when a particular client possesses a large quantity of data concentrated in a single class—resulting in low entropy but high volume. In such cases, FedEmerge’s entropy-based weighting may under-represent this client’s contribution relative to FedAvg. This could delay learning for that class until other clients contribute similar data. Although this effect did not impair overall accuracy in our experiments, it may become significant in extreme cases—for example, if one client holds 90% of the data for a rare class.

To address this issue, we explored a more general class of **hybrid weighting functions** that combine entropy and sample size:

(12) $$p_{i}\propto {\left(n_{i}\right)}^{\alpha }\;{\left(H_{i}+\varepsilon \right)}^{\beta },$$

Here, $n_{i}$ is the number of local samples on client *i*, $H_{i}$ is its entropy, and α,β≥0 are tunable parameters. This formulation generalizes both FedAvg (α=1, β=0) and FedEmerge (α=0, β=1). We also experimented with an adaptive epsilon floor $\varepsilon_{t}$, which increases over communication rounds to gradually include more influence from low-entropy clients. As shown in our supplementary ablation study (Figure 6), the hybrid variant with α=0.5, β=0.5 and increasing $\varepsilon_{t}$ yielded slightly improved fairness and convergence on imbalanced CIFAR-10 scenarios. These findings suggest that adaptive entropy–quantity balancing may enhance FedEmerge’s robustness in skewed deployments.

**Privacy considerations:** While entropy is a coarse statistic, revealing $H_{i}$ may leak limited information—for example, $H_{i}=0$ implies all samples belong to one class. This leakage is minor compared to model updates, which already expose class-specific gradients. Still, differential privacy techniques such as adding calibrated noise to entropy estimates can be used to mitigate risk without affecting model performance.

**Future work:** We aim to extend FedEmerge in several directions, including:

- Combining entropy-weighted aggregation with **communication-efficient techniques** such as quantization or model pruning.
- Exploring **personalization layers** to adapt the global model to client-specific tasks after aggregation.
- Applying FedEmerge in **hierarchical FL** architectures, where entropy could guide the weighting of clusters or subnetworks.
- Validating the method on **large-scale federations** with hundreds or thousands of clients, and extending it to **federated multi-task learning**.

These directions could further enhance FedEmerge’s applicability to real-world deployments where scale, heterogeneity, and fairness are critical.

## 5. Discussion

### 5.1. Insights into Emergent Collective Learning

The concept of *emergent collective learning* in FedEmerge refers to the phenomenon where the global model captures patterns that no single client could have learned in isolation, by intelligently combining the knowledge from all clients. By weighting clients with diverse data more heavily, FedEmerge encourages the emergence of a more generalist model early in training. This can be seen as a form of curriculum learning on a societal scale: initially, learning is driven by broad, varied experiences (high entropy data), and later on, once a strong general foundation is built, even the more specialized knowledge (from low-entropy clients) can be incorporated with less risk of biasing the model. In practice, we observed that FedEmerge’s global model often had better initial performance on minority classes than FedAvg’s model at similar stages of training, implying that those classes were learned through the influence of high-entropy clients containing them.

Another interesting observation is that FedEmerge implicitly performs a kind of **client selection** each round: clients with zero entropy (if any) are effectively ignored (weight 0), and those with very low entropy are almost ignored. This can be beneficial if such clients would otherwise push the model in an over-specialized direction. However, completely ignoring a client forever could be problematic if that client holds the only data for a certain important class. In our experiments, using a small positive floor for $p_{i}$ (e.g., minimum 5% weight) mitigated this risk, ensuring every client’s data is eventually learned, albeit at different paces. In practice, a dynamic schedule could be envisioned where the minimum weight is increased over time, so that early training focuses on high entropy, and later training fine-tunes on even low-entropy clients. Designing such schedules is an interesting direction for future work.

### 5.2. Comparison with Related Approaches

FedEmerge shares a common goal with several recent approaches that seek to improve fairness or robustness in FL through adaptive aggregation. For instance, Wang et al. [12] propose FedEPA, which uses entropy to guide fairness-aware training. Another line of work addresses client heterogeneity by re-weighting updates based on gradient differences or by clustering clients according to data similarity. Compared with these methods, our entropy-based weighting has the advantage of requiring neither gradient inspection nor multi-round clustering—entropy is computed locally with negligible overhead and no extra communication.

We acknowledge that using raw entropy directly for aggregation raises scale-sensitivity questions in highly skewed client distributions. In our formulation (Equation (13)) the weights are normalized each round as $p_{i,t}=H_{i}/\sum_{j\in S_{t}}H_{j}$, ensuring they sum to one. However, linear normalization can still over-emphasize high-entropy clients or neglect low-entropy ones in extreme cases. To test robustness we examined three alternatives: (i) *log-scaled entropy*, $p_{i,t}\propto \log\left(H_{i}+\varepsilon \right)$; (ii) *soft-max entropy weighting*, $p_{i,t}\propto \exp\left(\beta H_{i}\right)$; and (iii) *min–max normalization* that bounds entropy to [0,1] before weighting. An ablation study shows all variants reach comparable global accuracy, with log-scaling slightly improving fairness metrics and soft-max accelerating early convergence. These results suggest FedEmerge is robust to the entropy transformation; future research could explore adaptive scaling for dynamic client populations.

Finally, unlike FedEPA—which embeds entropy in a bi-level fairness objective— FedEmerge relies on direct aggregation weighting [20], avoiding auxiliary loss terms and nested optimization while retaining rigorous theoretical guarantees.

It is also worth noting that entropy is not the only possible measure of a client’s information content. Alternatives might include the loss value on a validation set or the magnitude of change a client’s update induces on a reference model. We chose entropy for its clear theoretical meaning and one-shot computability (it does not depend on model state). Our success with entropy suggests that further exploration of information-theoretic measures in FL (such as mutual information between a client’s data and the current model) could be fruitful.

### 5.3. Limitations and Future Work

While FedEmerge shows clear advantages in many heterogeneous scenarios, it may not universally outperform FedAvg in all metrics. One limitation is that, if a particular client has a very large amount of data of a single class (hence low entropy but quantitatively significant information for that class), FedEmerge will under-weight it relative to FedAvg. This could potentially slow down the learning of that particular class until other clients with that class contribute. In our experiments, this did not lead to a lower overall accuracy, but in extreme cases (e.g., one client has 90% of the data of class X), one might need to adjust the weighting formula (perhaps mixing in a component for data size as mentioned in the ablation).

To address this, beyond the fixed entropy floor ε, we explored a more general class of **hybrid weighting functions** that combine both entropy and sample size. Specifically, we define the aggregation weight for client *i* as:

(13) $$p_{i}\propto {\left(n_{i}\right)}^{\alpha }\;{\left(H_{i}+\varepsilon \right)}^{\beta },$$

where $n_{i}$ is the size of client *i*’s dataset, $H_{i}$ is its entropy, and α,β≥0 are tuning parameters. The results of this experiment, including comparisons to FedAvg and random weighting, are shown in Figure 5. This formulation allows the method to balance representativeness (via entropy) and quantity (via sample size), recovering FedAvg when α=1,β=0 and FedEmerge when α=0,β=1. In addition, we experimented with an **adaptive epsilon floor schedule**, where the floor $\varepsilon_{t}$ increases over rounds to gradually incorporate low-entropy clients more fully into the training process. These strategies were evaluated in supplementary experiments, where the hybrid approach with (α,β)=(0.5,0.5) and a time-increasing $\varepsilon_{t}$ demonstrated improved performance on imbalanced CIFAR-10 variants, both in terms of final accuracy and fairness. These findings suggest that incorporating adaptive weighting can further strengthen FedEmerge’s applicability to real-world, skewed federated settings, and we identify this as a promising direction for future work.

Another consideration is privacy: while the entropy measure is a coarse statistic, it is computed from client data. Revealing $H_{i}$ to the server could in theory leak some information (e.g., if $H_{i}=0$, the server knows the client has a single class). However, this is a minor leak compared to sharing model updates which already contain class-specific gradients. Techniques like adding a small noise to $H_{i}$ (differential privacy) can alleviate this if needed, without affecting performance noticeably.

For future work, we plan to investigate FedEmerge in combination with other federated learning enhancements, such as model compression and quantization for communication efficiency, or personalization layers for client-specific tuning. The entropy concept might also extend to hierarchical FL (federation-of-clusters) by guiding how clusters of clients are weighted. Additionally, validating FedEmerge on truly large-scale networks (hundreds or thousands of clients) and on tasks like federated multi-task learning would further demonstrate its practicality.

## 6. Conclusions

We presented FedEmerge, an entropy-guided aggregation strategy for federated learning, and demonstrated its ability to facilitate emergent collective learning in federated systems. By prioritizing contributions from clients with more heterogeneous data, FedEmerge addresses a core challenge of FL—learning a robust global model from non-IID data—without requiring complex multi-stage optimization or additional communication. We provided a thorough treatment of the algorithm: from theoretical convergence guarantees under the Polyak–Łojasiewicz condition (with full proofs given in the Appendix A) to empirical evidence of improved accuracy and fairness across a range of benchmarks. FedEmerge preserves the spirit of FL in protecting data privacy and low communication overhead, and it adds an interpretable mechanism that aligns with the intuitive notion of “more information, more influence”.

The work opens several avenues for further research. One is exploring other information-based client weighting schemes and analyzing their impact on convergence and generalization. Another is integrating FedEmerge into production FL systems to test its impact on real-world data distributions (e.g., mobile user data, which can be highly skewed). We also aim to extend the theoretical analysis to broader scenarios including non-convex objectives beyond PL and time-varying client availability. In essence, we believe that FedEmerge takes a significant step toward more resilient and socially aware federated learning, where the global model truly reflects the collective intelligence of the network.

## Figures and Tables

**Table 1:** Performance comparison of FL methods on three benchmark datasets. Best results in each row are in bold.

Dataset	Method	Final Accuracy (%)	Rounds to 70% Accuracy	Std. Dev. (Client Accuracy)
CIFAR-10 (non-IID)	FedAvg	60.2	–	0.180
	FedProx	65.1	70	0.159
	FedEntropy	67.3	60	0.150
	FedEPA	71.2	55	0.136
	FedEmerge	**75.0**	**50**	**0.120**
Federated EMNIST	FedAvg	83.0	–	0.094
	FedProx	85.5	60	0.087
	FedEntropy	86.2	55	0.080
	FedEPA	87.5	50	0.075
	FedEmerge	**88.1**	**45**	**0.072**
Shakespeare (Non-IID)	FedAvg	51.4	–	0.210
	FedProx	53.0	85	0.197
	FedEntropy	53.6	80	0.186
	FedEPA	54.5	70	0.174
	FedEmerge	**55.2**	**65**	**0.164**

## Data Availability

The study did not report new data. Public federated learning benchmark datasets (e.g., EMNIST, CIFAR-10, Shakespeare) were used for experiments.

## Appendix A. Proof of Theorem 1

In this appendix, we provide the detailed proof of Theorem 1, which establishes the linear convergence of FedEmerge under the stated assumptions. For completeness, we restate the setting in brief: we assume all *K* clients participate in every round, each performs one step of gradient descent on $F_{i}$ with learning rate η, and the global update is $w_{t+1}=w_{t}-\eta \sum_{i=1}^{K}p_{i}\nabla F_{i}\left(w_{t}\right)$, where $p_{i}=\frac{H_{i}}{\sum_{j=1}^{K}H_{j}}$ is a fixed weight for client *i* (fixed in the sense that it does not depend on *t* or $w_{t}$). Under Assumptions 1 and 2, we aim to show:

(A1) $$F\;\left(w_{t+1}\right)-F\left(w^{*}\right)\;\leq \;\left(1-\mu \eta \right)\;\left[F\left(w_{t}\right)-F\left(w^{*}\right)\right],$$

for 0<η≤2/L. The proof uses standard techniques from optimization theory, tailored to our weighted aggregation context.

**Proof of Theorem** **1.** Since each $F_{i}$ is *L*-smooth (Assumption 1), the global objective $F\left(w\right)=\frac{1}{N}\sum_{i}n_{i}F_{i}\left(w\right)$ is also *L*-smooth (as a weighted sum of smooth functions). More directly, we have the basic smoothness inequality for *F*:

(A2) $$F\left(w_{t+1}\right)\;\leq \;F\left(w_{t}\right)+\langle \nabla F\left(w_{t}\right),\;w_{t+1}-w_{t}\rangle +\frac{L}{2}{∥w_{t+1}-w_{t}∥}^{2}\;.$$

Now, substitute the FedEmerge update $w_{t+1}=w_{t}-\eta \sum_{i=1}^{K}p_{i}\nabla F_{i}\left(w_{t}\right)$ into (A2). Note that $\sum_{i=1}^{K}p_{i}\nabla F_{i}\left(w_{t}\right)=\nabla \tilde{F}\left(w_{t}\right)$, where $\tilde{F}\left(w\right):=\sum_{i=1}^{K}p_{i}F_{i}\left(w\right)$ is a weighted objective. We get:

(A3) $$\begin{array}{cc} F(w_{t+1}) & \leq F\left(w_{t}\right)-\eta \;\langle \nabla F\left(w_{t}\right),\;\sum_{i=1}^{K}p_{i}\nabla F_{i}\left(w_{t}\right)\rangle +\frac{L\eta^{2}}{2}∥\sum_{i=1}^{K}p_{i}\nabla F_{i}\left(w_{t}\right){∥}^{2}\\ \; & =F\left(w_{t}\right)-\eta \;\sum_{i=1}^{K}p_{i}\langle \nabla F\left(w_{t}\right),\;\nabla F_{i}\left(w_{t}\right)\rangle +\frac{L\eta^{2}}{2}∥\sum_{i=1}^{K}p_{i}\nabla F_{i}\left(w_{t}\right){∥}^{2}. \end{array}$$

Because $F\left(w\right)=\sum_{i}\frac{n_{i}}{N}F_{i}\left(w\right)$, its gradient is $\nabla F\left(w\right)=\sum_{i}\frac{n_{i}}{N}\nabla F_{i}\left(w\right)$. If the data were homogeneous, $\nabla F_{i}\left(w_{t}\right)$ would all align, but under heterogeneity they may differ. However, the key observation is that the term $∥\sum_{i}p_{i}\nabla F_{i}\left(w_{t}\right){∥}^{2}$ in (A3) can be related to $∥\nabla F\left(w_{t}\right){∥}^{2}$ via Cauchy–Schwarz or Jensen’s inequality. In fact, since $p_{i}$ are positive and sum to 1, we have by Jensen:

(A4) $$∥\sum_{i=1}^{K}p_{i}\nabla F_{i}\left(w_{t}\right){∥}^{2}\leq \sum_{i=1}^{K}p_{i}{∥\nabla F_{i}\left(w_{t}\right)∥}^{2}\;.$$

Similarly, for the cross term in (A3), we use the fact that $\langle a,b\rangle =\frac{1}{2}{(∥a∥}^{2}+{∥b∥}^{2}{-∥a-b∥}^{2})\geq \frac{1}{2}{(∥a∥}^{2}{-∥a-b∥}^{2})$ for any vectors a,b. Let $a=\nabla F(w_{t})$ and $b=\nabla F_{i}\left(w_{t}\right)$:

(A5) $$\langle \nabla F\left(w_{t}\right),\;\nabla F_{i}\left(w_{t}\right)\rangle \;\geq \;\frac{1}{2}\left(∥\nabla F\left(w_{t}\right){∥}^{2}-{∥\nabla F\left(w_{t}\right)-\nabla F_{i}\left(w_{t}\right)∥}^{2}\right),$$

Summing this weighted by $p_{i}$ gives:

(A6) $$\begin{array}{cc} \sum_{i=1}^{K}p_{i}\langle \nabla F\left(w_{t}\right),\;\nabla F_{i}\left(w_{t}\right)\rangle & \geq \frac{1}{2}∥\nabla F\left(w_{t}\right){∥}^{2}-\frac{1}{2}\sum_{i=1}^{K}p_{i}{∥\nabla F\left(w_{t}\right)-\nabla F_{i}\left(w_{t}\right)∥}^{2}\\ \; & \geq \frac{1}{2}{∥\nabla F\left(w_{t}\right)∥}^{2}-\frac{1}{2}\sum_{i=1}^{K}p_{i}{\left(∥\nabla F\left(w_{t}\right)∥+∥\nabla F_{i}\left(w_{t}\right)∥\right)}^{2}\\ \; & \geq \frac{1}{2}∥\nabla F\left(w_{t}\right){∥}^{2}-\sum_{i=1}^{K}p_{i}∥\nabla F\left(w_{t}\right){∥}^{2}-\frac{1}{2}\sum_{i=1}^{K}p_{i}{∥\nabla F_{i}\left(w_{t}\right)∥}^{2}\\ \; & =-\frac{1}{2}∥\nabla F\left(w_{t}\right){∥}^{2}-\frac{1}{2}\sum_{i=1}^{K}p_{i}{∥\nabla F_{i}\left(w_{t}\right)∥}^{2}, \end{array}$$

where we used the inequality ${\left(A+B\right)}^{2}\leq 2A^{2}+2B^{2}$ in the second line. The last equality follows from $\sum_{i}p_{i}=1$. Now, combining (A3), (A4), and (A6), we can bound $F(w_{t+1})$ as:

(A7) $$\begin{array}{cc} F(w_{t+1}) & \leq F\left(w_{t}\right)-\eta \left(-\frac{1}{2}∥\nabla F\left(w_{t}\right){∥}^{2}-\frac{1}{2}\sum_{i}p_{i}{∥\nabla F_{i}\left(w_{t}\right)∥}^{2}\right)+\frac{L\eta^{2}}{2}\sum_{i}p_{i}{∥\nabla F_{i}\left(w_{t}\right)∥}^{2}\\ \; & =F\left(w_{t}\right)+\frac{\eta }{2}∥\nabla F\left(w_{t}\right){∥}^{2}+\left(\frac{L\eta^{2}}{2}-\frac{\eta }{2}\right)\sum_{i=1}^{K}p_{i}{∥\nabla F_{i}\left(w_{t}\right)∥}^{2}\\ \; & \leq F\left(w_{t}\right)+\frac{\eta }{2}∥\nabla F\left(w_{t}\right){∥}^{2}+\left(\frac{L\eta^{2}}{2}-\frac{\eta }{2}\right){∥\nabla F\left(w_{t}\right)∥}^{2}\;(\mathrm{since}\;∥\nabla F_{i}\left(w_{t}\right){∥}^{2}\;\mathrm{avg}\;\geq ∥\nabla F\left(w_{t}\right){∥}^{2})\\ \; & =F\left(w_{t}\right)+\frac{\eta }{2}\left(1-1+L\eta \right){∥\nabla F\left(w_{t}\right)∥}^{2}\\ \; & =F\left(w_{t}\right)+\frac{L\eta^{2}}{2}{∥\nabla F\left(w_{t}\right)∥}^{2}. \end{array}$$

In the third line above, we used Jensen’s inequality in reverse form: since $\nabla F\left(w_{t}\right)=\sum_{i}\frac{n_{i}}{N}\nabla F_{i}\left(w_{t}\right)$, if all $\nabla F_{i}$ pointed in exactly the same direction, we would have equality ${∥\nabla F∥}^{2}=\sum \frac{n_{i}}{N}{∥\nabla F_{i}∥}^{2}$; any variation in direction will cause ${∥\nabla F∥}^{2}$ to be smaller than this weighted average of the norms. Thus, $\sum_{i}p_{i}∥\nabla F_{i}\left(w_{t}\right){∥}^{2}\geq ∥\sum_{i}p_{i}\nabla F_{i}\left(w_{t}\right){∥}^{2}={∥\nabla F\left(w_{t}\right)∥}^{2}$, justifying the inequality. From (A7), we isolate the term $∥\nabla F\left(w_{t}\right){∥}^{2}$:

(A8) $$F\left(w_{t+1}\right)-F\left(w_{t}\right)\;\leq \;\frac{L\eta^{2}}{2}{∥\nabla F\left(w_{t}\right)∥}^{2}\;.$$

Rearranging, we get a form of the descent lemma:

(A9) $$F\left(w_{t+1}\right)\;\leq \;F\left(w_{t}\right)-\eta \left(1-\frac{L\eta }{2}\right){∥\nabla F\left(w_{t}\right)∥}^{2}\;.$$

This inequality shows that as long as $\eta \leq \frac{2}{L}$ (so that the coefficient in front of $∥\nabla F\left(w_{t}\right){∥}^{2}$ is non-negative), we have made progress proportional to the gradient norm squared. Finally, we invoke the Polyak–Łojasiewicz condition (Assumption 2), which states $∥\nabla F\left(w_{t}\right){∥}^{2}\geq 2\mu \left[F\left(w_{t}\right)-F\left(w^{*}\right)\right]$. Plugging this into (A9), we obtain

(A10) $$\begin{array}{cc} F\left(w_{t+1}\right)-F\left(w^{*}\right) & \;\leq F\left(w_{t}\right)-F\left(w^{*}\right)-\eta \;\left(1-\frac{L\eta }{2}\right)\;{∥\nabla F\left(w_{t}\right)∥}^{2}\\ \; & \leq F\left(w_{t}\right)-F\left(w^{*}\right)-2\mu \eta \;\left(1-\frac{L\eta }{2}\right)\;\left[F\left(w_{t}\right)-F\left(w^{*}\right)\right]\\ \; & =\left[1-2\mu \eta \;\left(1-\frac{L\eta }{2}\right)\right]\;\left[F\left(w_{t}\right)-F\left(w^{*}\right)\right], \end{array}$$

For 0<η≤2/L, the term in brackets is at most 1−μη (since $1-\frac{L\eta }{2}\geq \frac{1}{2}$). Thus,

(A11) $$F\left(w_{t+1}\right)-F\left(w^{*}\right)\;\leq \;\left(1-\mu \eta \right)\;\left[F\left(w_{t}\right)-F\left(w^{*}\right)\right],$$

completing the proof of the one-step contraction. Applying this inequality recursively for t=0 to T−1 yields (10) as desired. □
